# Detecting Distress in Cognitively Impaired People to Prevent Suffering: Protocol for an Observational Feasibility Study of a Radar-Based Technology Augmented with Photoplethysmographic Sensors and Audio Signals (SURREAL)

**DOI:** 10.3390/s26144484

**Published:** 2026-07-15

**Authors:** Christopher Boehlke, Fabian Buergi, Jens Eckstein, Marc Stawiski, Simone Hemm, Wolfgang Hasemann, Jan Gaertner

**Affiliations:** 1 Palliative Care Center, Bethesda Spital AG, 4052 Basel, Switzerland; fabian.buergi@usb.ch (F.B.);; 2 Department of Clinical Research, University Hospital Basel, University of Basel, 4031 Basel, Switzerland; 3 Department Digitalization & ICT, University Hospital Basel, 4031 Basel, Switzerland; jens.eckstein@usb.ch; 4 Department of Internal Medicine, University Hospital Basel, 4031 Basel, Switzerland; 5 Institute for Medical Engineering and Medical Informatics, School of Life Sciences, University of Applied Sciences and Arts Northwestern Switzerland, 4132 Muttenz, Switzerlandsimone.hemm@fhnw.ch (S.H.); 6 Institute of Nursing Science, University of Basel, 4031 Basel, Switzerland; w.hasemann@unibas.ch

**Keywords:** palliative care, digital health, radar, wearable devices, distress detection, dyspnea

## Abstract

**Background**: With the increasing prevalence of multimorbidity, the demand for palliative and end-of-life care is expected to rise substantially in the coming decades. Digital health technologies may enable automated detection of clinically relevant distress, including symptoms such as pain, breathlessness (dyspnea), anxiety/panic, nausea, and agitation. Remote detection of such distress in cognitively impaired patients who are unable to reliably call for help could enable timely intervention when patients are unattended. **Methods**: This observational feasibility study will collect multimodal data from a non-invasive sensor system consisting of 3D radar, a photoplethysmographic sensor (wearable), and a microphone. Sensor data will be linked to distress events identified by nurses or physicians during routine clinical care using structured proxy assessments. Adults (≥18 years) admitted to the Palliative Care Center Basel who are unable to reliably call for help due to cognitive impairment will be included based on written informed consent provided by a legal proxy. **Aim**: The aim of this study is to evaluate the feasibility of multimodal sensor-based monitoring for detecting clinician-identified distress events and to explore associations between sensor-derived variables and distress, informing future validation studies and the development of automated detection approaches in palliative care.

## 1. Introduction

Palliative care is the active holistic care of individuals across all ages with serious health-related suffering due to severe illness and especially of those near the end of life [1]. In patients who are able to communicate, relief of suffering is primarily achieved through the identification of distress by asking patients about their symptoms. This is typically facilitated using standardized patient-reported outcome measures presented as brief questionnaires assessing common distressing symptoms such as pain, dyspnea, anxiety/panic, and agitation [2,3]. However, these instruments cannot be applied in cognitively impaired patients who are unable to communicate their needs reliably.

In this present study, distress is defined as clinically relevant somatic and psychological symptoms such as pain, dyspnea, nausea, anxiety/panic, and agitation, which may lead to substantial psychological suffering [4]. Pain and breathlessness are among the most common sources of distress in patients dying in hospital [5]. Nearly half of patients with cancer experience moderate to severe pain, with prevalence increasing toward the end of life [6,7]. Breathlessness is also highly prevalent, affecting patients with advanced cancer as well as terminal cardiac or pulmonary disease [8]. Among patients receiving end-of-life care, the proportion experiencing severe breathlessness increases from 10% to 26% [9].

Cognitive impairment is highly prevalent in palliative care populations. Delirium occurs in 14–85% of patients, and up to 90% of patients experience some degree of cognitive impairment before death [10,11]. Together with demographic changes and increasing multimorbidity, this is expected to substantially increase the demand for palliative and end-of-life care in the coming decades [12,13,14]. These developments pose a significant challenge for healthcare systems, particularly due to the growing burden on clinical staff responsible for continuous patient monitoring [15].

In patients unable to self-report symptoms, the current reference standard for identifying distress is clinical assessment by experienced nurses or physicians [16]. However, timely recognition of sudden distress episodes would require continuous bedside observation, which is rarely feasible in routine care. Consequently, episodes of distress may remain unrecognized for prolonged periods.

Digital health technologies, defined as the use of information and communication technologies to manage illness and health risks and to promote well-being, may help address this challenge [17]. Advances in wearable devices and contactless sensor systems increasingly enable continuous measurement of physiological and behavioral parameters potentially associated with symptom burden [18,19,20,21,22,23,24,25]. Nevertheless, it remains unclear which objectively measurable sensor-derived parameters are reliably associated with distress events in cognitively impaired palliative care patients who are unable to call for help.

## 2. Experimental Design

**Figure 1:**
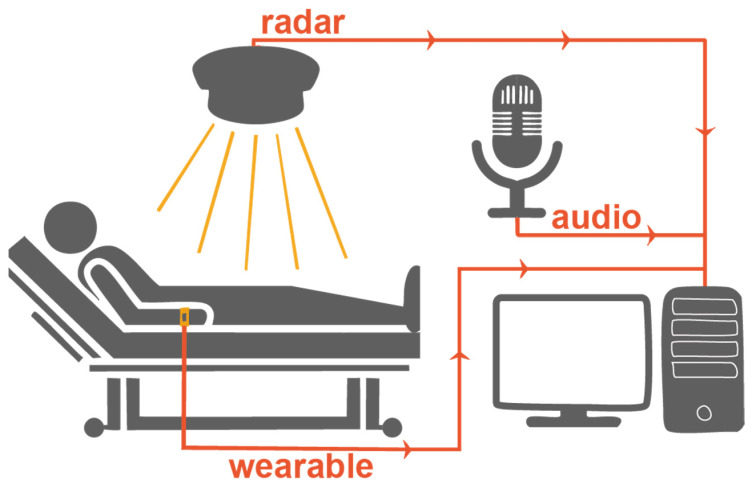
Experimental setup: input from radar, microphone and wearable.

This observational feasibility study aims to assess the feasibility of multimodal sensor-based monitoring and to explore associations between sensor-derived parameters and clinician-identified distress events. The multimodal sensor comprises a 3D radar, a photoplethysmographic sensor (wearable), and a microphone (Figure 1).

## 3. Materials and Equipment

### 3.1. Sensor System

The sensor system will consist of three main components:

- An established radar system (Qumea);
- A wearable with a photoplethysmographic sensor;
- A microphone enabling extraction of acoustic features potentially associated with distress.

The patient’s radar, photoplethysmographic, and audio signals will be recorded and logged with specific timestamps. The obtained signals and physiological items to be recorded will include:

- Radar raw signals (“point cloud”) (Qumea);
- Body movement index (between 0 and 1) (Qumea);
- Respiratory rate (Qumea);
- Heart rate and heart rate variability (wearable);
- Acoustic biomarkers, including sound pressure levels, spectral centroids, the duration and cadence of non-verbal vocalizations and other paralinguistic features.

### 3.2. Output of 3D Radar Sensor (Qumea)

The Qumea system is a ceiling-mounted 60 GHz 3D radar sensor enabling contactless monitoring of patient movement and respiratory activity. Radar signals generate spatial motion data (“point cloud”) from which movement intensity (motility index, 0–1 scale) and respiratory rate are derived. The sensor does not capture optical images and does not allow visual identification of individuals. Data are time-stamped and stored in pseudonymized form using technical identifiers (room and bed number). Radar-derived parameters are analyzed as continuous variables within predefined observation windows preceding clinical distress assessments.

### 3.3. Output of Photoplethysmographic Sensors (Wearables)

Wearable photoplethysmographic sensors enable continuous, non-invasive measurement of physiological parameters, particularly heart rate and heart rate variability [26]. Signal quality may be affected by motion artifacts, especially when devices are worn on the wrist. Previous studies have reported approximately 50% usable signal segments under ambulatory conditions; as the study population is predominantly bedridden, a higher proportion of analyzable data is expected. Devices require intermittent removal for charging approximately every four days. Wearable monitoring is minimally intrusive and does not interfere with routine care.

### 3.4. Audio Analysis and Acoustic Biomarker

To complement physiological and movement data, an acoustic recording system will capture vocalizations potentially associated with distress (e.g., moaning or gasping). A VT506Mobile microphone (VT SWITZERLAND AG, Zuerich, Switzerland) (40 Hz–20 kHz) will be installed at the bedside. Audio signals will be recorded at a 48 kHz sampling rate and 24-bit depth and stored as uncompressed WAV files. The analysis will include established acoustic descriptors such as sound pressure level and spectral centroid, complemented by additional features characterizing the spectral distribution, temporal dynamics, and variability of non-verbal vocalizations. These include measures capturing the duration and cadence of vocal events as well as broader paralinguistic attributes reflecting voice quality and expressivity. These parameters may reflect physiological strain or acute agitation and will be used to derive candidate acoustic biomarkers associated with distress.

Raw audio signals are temporarily stored in encrypted form to enable signal processing and extraction of acoustic features. Access to raw audio data is restricted to authorized technical personnel involved in signal processing and quality control. To minimize privacy risks, only audio data from the 10 min period preceding a distress or non-distress event will be retained for processing and analysis; all other audio recordings will be automatically deleted. Raw audio data will be retained for 10 years and deleted thereafter. They will not be used to analyze speech content, conversations, or identify individuals.

To further protect privacy, distress and non-distress events will only be annotated when no visitors or relatives are present in the room. In addition, a clearly visible notice will be placed on the patient room door indicating that audio recording is in progress as part of a research study. Healthcare professionals working on the unit will receive specific information and training regarding the audio component of the study, including the fact that recordings are used exclusively for extraction of non-verbal acoustic features and that speech content is neither analyzed nor evaluated. These measures are intended to minimize the risk of capturing sensitive third-party information while allowing investigation of acoustic markers associated with distress.

### 3.5. Distress Annotation

Here, distress is operationally defined as the presence of clinically relevant symptoms that typically require medical or nursing intervention in specialist palliative care. Symptom domains include pain, dyspnea, anxiety/panic, nausea, and agitation. The definition focuses on observable and treatment-relevant distress to ensure the feasibility of this sensor array, data sharing and evaluation methods in cognitively impaired patients unable to reliably self-report (Table 1).

Because many included patients will not be able to provide valid self-report, symptom severity will be documented using proxy-rated ESAS [27] assessments completed by trained nurses or physicians based on structured clinical observation during routine care. The reference standard consists of clinician-identified distress events documented during routine care using predefined symptom categories and severity ratings.

Distress events will be documented at routine clinical visits, expected approximately once per hour. For each observation, clinicians will record the presence or absence of distress, symptom domain, and severity (ESAS 0–10 scale). To minimize interference with routine care, two documentation approaches will be evaluated during an implementation phase: a tablet-based application and a paper-based time-stamped recording system. The method with higher acceptability among healthcare staff will be used in the main study.

A multiprofessional training workshop will be conducted to promote a shared understanding of clinically relevant distress events and to reduce interindividual variability in assessments. All participating nurses and physicians will receive structured training using written definitions, examples, and case vignettes. Regular team discussions during the implementation phase will be used to clarify ambiguous cases and maintain consistency across raters.

### 3.6. Synchronization of Data

Accurate temporal alignment of sensor data and clinical annotations is essential. Data from the radar system (Qumea), wearable sensors, and the audio recording system will be transferred to secure study-specific storage environments and time-stamped using the Network Time Protocol (NTP) to a common institutional time server. Minor discrepancies between timestamps may occur due to network latency, device-specific processing delays, or clock drift.

During an initial implementation phase, synchronization accuracy will be evaluated by quantifying timestamp differences between systems. The acceptable temporal deviation between data streams is defined as ≤5 s. If deviations exceed this threshold, the affected data streams will be corrected where possible or flagged and excluded from time-sensitive analyses.

## 4. Detailed Procedure

### 4.1. Inclusion and Exclusion Criteria

A total of 30 patients receiving specialized palliative care will be included (compare Section 4.5). Patients in this setting are frequently admitted due to a clinically relevant symptom burden [28], and a substantial proportion experience cognitive impairment during their disease trajectory [29]. Patients in the actively dying phase will be excluded, as physiological and behavioral patterns during imminent death may substantially differ from those in other palliative care phases and could confound the interpretation of distress-related signals.

#### 4.1.1. Inclusion Criteria

- Admission or transfer to the Palliative Care Center Basel;
- Age ≥ 18 years;
- Cognitive impairment with inability to reliably call for help;
- Written informed consent provided by a legal proxy.

#### 4.1.2. Exclusion Criteria

- Inability to obtain proxy consent due to language barriers;
- Absence of a legal proxy;
- Persistent unconsciousness, Cheyne–Stokes respiration, terminal secretions, or expected death < 72 h.

### 4.2. Patient Assessments

Data collection will begin upon admission to a room equipped with the Qumea radar system. A wearable sensor will be placed on the patient to enable continuous 24/7 recording of physiological parameters. A bedside microphone will be installed to enable acoustic recording throughout the monitoring period. During routine care, nurses and physicians will assess the presence or absence of distress events.

At inclusion and weekly thereafter, cognitive status and delirium will be assessed using the Modified Confusion Assessment Method for the Emergency Department (mCAM-ED) [30] and the Delirium Rating Scale Revised-98 (DRS-R-98) [31]. Information on pre-hospital functional status will be obtained from relatives using the Functional Independence Measure (FIM) [32]. Demographic and clinical data, including age, sex, diagnosis, body mass index (BMI), height, weight (estimated if necessary), and prior symptom burden, will be extracted from the hospital information system and entered into the study REDCap^®^ database (Table 2).

During routine care visits, nurses or physicians will document whether a distress event requiring clinical intervention is present or absent. Although multiple individuals may enter the patient’s room (e.g., therapists, visitors, or cleaning staff), only trained nurses and physicians will perform distress assessments to ensure consistency of the reference standard. Participants will be observed throughout their entire hospital stay, with an expected median duration of approximately 7 days.

### 4.3. Data Processing

Data collected during each 10 min observation window will be aggregated into a single summary value for each predictor. Trends within the interval will not be assessed. For respiration rate, heart rate, and movement, the mean values will be taken across all sensor measurements for the interval. For ordinal variables (SV), we will take the median value of the numbered values of categories. If the median value is a non-integer value, it will be rounded to the nearest integer. For heart rate variability, the standard deviation of all heart rate measures will be calculated. For audio recordings, a composite acoustic index will be derived by calculating the mean values of primary and exploratory biomarkers (e.g., jitter, shimmer, and spectral distribution) across all validated vocalization segments identified within the 10 min interval.

Missing data between measured points in the interval will be midpoint interpolated. Missing data at the start and end of the interval will be excluded. Intervals in which one or more variables have insufficient data (available for less than 60% of the time points) will be excluded from the analysis. Distress events and other clinical annotations that are missing or incomplete will not be imputed; observation windows lacking the required outcome information will be excluded from analyses involving that outcome.

### 4.4. Statistical Analysis and Methodology

The full analysis set will include all patients meeting inclusion and exclusion criteria. If patients withdraw from the study (e.g., revoking consent), data up to the time of withdrawal will be used if possible. People entering the patient’s room come from different disciplines and professions [33] and include nursing staff, medical staff, other clinical staff (e.g., physical therapists), cleaning personnel, and personal visitors. Only registered nurses and physicians will be asked to decide if there is a distress episode in need of a medical intervention. When they check in on a patient, they will document whether a distress event occurs or confirm the absence of distress (non-distress event). Only data collected during the 10 min interval prior to independent observation and recording of ‘distress’ or ‘no distress’ by a physician or nurse will be used.

A restricted analysis set will include intervals with complete data for all six candidate predictor variables and at least one distress and one non-distress observation per patient. This restriction aims to support stable parameter estimation while acknowledging potential enrichment of patients with higher distress frequency.

To reduce physiological heterogeneity within the non-distress reference category, non-distress observation windows will be sub-classified in exploratory analyses according to observable behavioral state, where available. Categories will include quiet wakefulness, sleep/resting state, and active wakefulness/movement.

#### 4.4.1. Primary Objective

**Figure 2:**
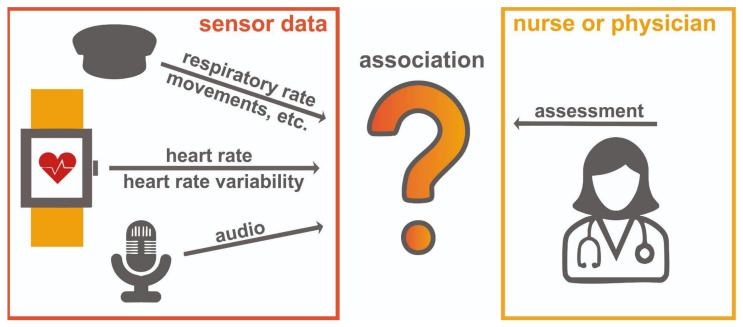
Association between sensor data and nurse- and physician-led assessments (gold standard).

The primary objective is to explore potential associations between six candidate sensor-derived variables and clinician-identified distress events (Figure 2):

- Respiratory rate (RR; radar-derived);
- Movement intensity (MV; radar-derived);
- Mean heart rate (HRM; wearable-derived);
- Heart rate variability (HRV; wearable-derived);
- Sound pressure level (SPL; audio-derived);
- Spectral centroid (SC, audio-derived).

The AUC/C-statistic will be used as a descriptive measure of the potential discriminatory ability of the investigated sensor-derived variables. As this is a feasibility study, these analyses are exploratory and hypothesis-generating and are intended to inform the design and sample size considerations of future validation studies rather than establish predictive performance. The primary mixed-effects logistic regression model will be repeated with behavioral state included as an additional fixed-effect covariate. Furthermore, where sample size permits, analyses will be repeated after restricting the reference category to homogeneous non-distress periods (e.g., sleep/resting state or quiet wakefulness). These analyses will assess the robustness of associations between sensor-derived variables and distress events to potential state-related confounding.

#### 4.4.2. Secondary Analysis

Multiple secondary hypotheses will be investigated in an exploratory context. As this is an initial study exploring the subject, these exploratory hypotheses are subject to change. Secondary analyses will include:

- Individual associations between each predictor variable and distress event;
- Correlations between predictor variables;
- Variation in associations between subgroups (e.g., cancer status, age > 65 years, sex);
- Associations between symptom type or severity and predictor variables;
- Agreement between nurse- and physician-based distress assessments;
- Influence of alternative time windows for prediction;
- Between-patient variability in distress incidence;
- Typical distress expression (informal caregivers’ view).

#### 4.4.3. Feasibility Endpoints

Feasibility endpoints include recruitment rate, proportion of eligible patients enrolled, proportion of completed observation days, completeness of sensor-derived data streams, and frequency of study-related reasons for discontinuation (e.g., proxy withdrawal). These endpoints will be reported descriptively and will inform the design of a future validation study.

### 4.5. Feasibility Considerations and Sample Size

As a feasibility study, all analyses evaluating associations between sensor-derived variables and distress events are exploratory and hypothesis-generating. The primary aim is to estimate effect sizes, assess data completeness, and evaluate the feasibility of synchronized multimodal data collection. Results will inform the design of future validation studies and should not be interpreted as definitive evidence of predictive performance.

Several parameters relevant for sample size estimation are uncertain. Estimates for distress incidence and number of observations per patient were based on the clinical experience of the study team. Assumptions regarding effect sizes were informed by Ritsert et al. [34], who reported standardized mean differences (SMD, Cohen’s d) of approximately 0.2–0.25 for heart rate and respiratory rate between stressful and non-stressful conditions. Comparable effect sizes were conservatively assumed for movement and acoustic variables.

Simulations were conducted assuming six candidate predictor variables, with SMDs between distress and non-distress observations ranging from 0.225 to 0.35. Additional pragmatic and conservative assumptions included 84 observations per patient (approximately 12 per day over 7 days), a baseline distress incidence of 0.1 (≈1.2 events per day), and between-patient variability in distress probability (log-odds SD = 0.5). Predictor variables were simulated using Gaussian distributions (SD = 1), with mean differences reflecting the assumed effect sizes. The assumed distress incidence rate of 0.1 was based on clinical experience within the study setting rather than formal pilot data, reflecting the absence of published estimates for this specific patient population and endpoint definition.

Under these assumptions, simulations suggested that approximately 25 participants would provide sufficient data to explore potential associations between sensor-derived variables and distress events. Allowing for attrition, a total sample size of 30 patients was considered appropriate for this feasibility study.

### 4.6. Schedule and Milestones

The study is planned over 24 months (Table 3). The first three months will focus on technical implementation, including setup of the data management system (REDCap^®^ 16.0.37), testing of the distress annotation procedures, and evaluation of synchronization between sensor systems. During this phase, a multiprofessional workshop will be conducted to establish a shared understanding of clinically relevant distress events and to standardize assessment procedures (M1).

Recruitment will take place from months 4 to 21. Eligible patients will be identified during routine care, and informed consent will be obtained from legal proxies by GCP-trained physicians (M2: first patient in; M3: last patient out).

Data analysis will be performed during the final three months, focusing on evaluation of feasibility outcomes and exploratory assessment of associations between sensor-derived variables and distress events. Results will be prepared for publication by the interdisciplinary study team (M4).

### 4.7. Patient and Public Involvement

During proposal development, we involved an EUPATI-trained patient representative to ensure alignment with patient needs and perspectives. His input informed several key elements, including the decision to conduct a multiprofessional workshop to reduce interindividual variability in distress assessments, in which he will participate to contribute both patient and caregiver perspectives. Acknowledging that patients express distress in diverse ways, he also recommended incorporating informal caregivers’ views on typical distress expression, which has been included as a secondary analysis. In addition, he advised on the feasibility of the recruitment strategy and will support the development of informational materials for family members. Finally, the patient representative will contribute to the analysis and dissemination of the results to patient communities and stakeholders, supporting the translation of findings into palliative care practice.

## 5. Discussion

Early recognition of distress is crucial in patients with advanced disease, particularly in those unable to reliably communicate their needs. Timely identification of clinically relevant distress may enable prompt therapeutic interventions for symptoms such as pain, dyspnea, anxiety, or agitation, thereby reducing avoidable suffering. This study aims to generate feasibility data on whether multimodal sensor-derived parameters are associated with clinician-identified distress events in specialized palliative care.

We expect the results to provide preliminary evidence on the usability, data quality, and clinical relevance of combining radar-based movement analysis, wearable-derived physiological parameters, and acoustic features for distress detection. The findings will inform the design of future validation studies and help determine which sensor-derived variables show the most promising association with clinically meaningful distress events.

Several limitations should be acknowledged. Distress assessments are based on clinician observations and therefore remain subject to inter-rater variability. Furthermore, behavioral state, comorbidities, medication effects, and the heterogeneous nature of distress symptoms may influence sensor-derived parameters. These factors will be explored where feasible and will inform the design of future validation studies. Furthermore, continuous monitoring systems may be affected by environmental and physiological sources of signal drift, including changes in room conditions, patient repositioning, sensor displacement, variations in body position, and evolving clinical status. As a feasibility study, the present project primarily focuses on data acquisition, synchronization, and preliminary signal characterization rather than the development of drift compensation algorithms. Nevertheless, future validation studies may benefit from adaptive signal-processing [35] approaches designed to identify and correct for long-term variability in sensor signals, thereby improving robustness and generalizability of multimodal distress detection systems.

More broadly, this project contributes to the integration of digital health technologies into palliative care research by addressing a population that is often excluded from patient-reported outcome-based approaches. If feasibility is demonstrated, the findings will provide the foundation for future multicenter validation studies and the development of automated distress detection systems for cognitively impaired patients and help guide individualized supportive care strategies.

## Figures and Tables

**Table 1:** Candidate physiological correlates of distress. Arrow pointing up depicting increase; arrow pointing down decrease.

Parameter	Potential Indicator for
↑ Heart rate (tachycardia)	pain, dyspnea, anxiety, nausea
↓ Heart rate variability	pain
↑ Respiratory rate (tachypnea)	pain, dyspnea, anxiety, nausea
↑ Movements	pain, dyspnea, anxiety, nausea
Acoustic signals (amplitude, frequency)	pain, dyspnea, anxiety

**Table 2:** Data collection schedule. + = performed once at the indicated visit.

Day	−1	0	
Visit	Screening	Inclusion	Weekly visit
Informed consent		+	
Baseline characteristics		+	
Assessments		mCAM-ED DRS-R-98	mCAM-ED DRS-R-98
		continuous sensor-data output continuous distress annotation	

**Table 3:** Study timeline and milestones.

Months	1–3 ^1^	4–6 ^2^	7–9	10–12	13–15	16–18	19–21 ^3^	22–24
Preparatory phase	+							
Recruitment		+	+	+	+	+	+	
Analysis								+
Milestones	M1	M2					M3	M4

^1^ Implementation, annotation. ^2^ First patient in. ^3^ Last patient in/last patient out. M1 Workshop. M2 First patient in. M3 Last patient out. M4 Manuscript submission. X Activity performed during the indicated time period.

## Data Availability

No new data were created or analyzed in this study.

## Abbreviations

The following abbreviations are used in this manuscript:

AUC	Area under the curve
CRF	Case report form
DRS-R-98	Delirium Rating Scale Revised-98
ESAS	Edmonton Symptom Assessment System
FIM	Functional Independence Measure
GCP	Good Clinical Practice
HRM	Mean heart rate
HRV	Heart rate variability
mCAM-ED	Modified Confusion Assessment Method for the Emergency Department
MV	Movement intensity
RR	Respiratory rate
SD	Standard deviation
SMD	Standardized mean difference
SC	Spectral centroid
SPL	Sound pressure level
SV	Sound volume
